# Efficacy of a Mobile App-Based Intervention for Young Adults With Anxiety Disorders: A Randomized Clinical Trial

**DOI:** 10.1001/jamanetworkopen.2024.28372

**Published:** 2024-08-20

**Authors:** Jennifer N. Bress, Avital Falk, Maddy M. Schier, Abhishek Jaywant, Elizabeth Moroney, Monika Dargis, Shannon M. Bennett, Matthew A. Scult, Kevin G. Volpp, David A. Asch, Mohan Balachandran, Roy H. Perlis, Francis S. Lee, Faith M. Gunning

**Affiliations:** 1 Department of Psychiatry, Weill Cornell Medicine, New York, New York; 2 Ferkauf Graduate School of Psychology, Yeshiva University, Bronx, New York; 3 Penn Center for Health Incentives and Behavioral Economics, University of Pennsylvania, Philadelphia; 4 Perelman School of Medicine, University of Pennsylvania, Philadelphia; 5 Center for Quantitative Health, Massachusetts General Hospital, Boston, Massachusetts; 6 Department of Psychiatry, Harvard Medical School, Boston, Massachusetts; 7 Associate Editor, *JAMA Network Open*

## Abstract

**Importance:**

Anxiety disorders are prevalent and undertreated among young adults. Digital mental health interventions for anxiety are promising but limited by a narrow range of therapeutic components and low user engagement.

**Objective:**

To investigate the efficacy of and engagement with Maya, a scalable, self-guided, comprehensive mobile cognitive behavioral therapy (CBT) intervention with embedded engagement features, comparing the effects of 3 incentive conditions.

**Design, Setting, and Participants:**

This randomized clinical trial recruited young adults aged 18 to 25 years with anxiety disorders through online advertisements and outpatient psychiatry clinics at Weill Cornell Medicine. Enrollment was between June 16, 2021, and November 11, 2022. Data analysis was performed from December 21, 2022, to June 14, 2024.

**Intervention:**

Participants received a 6-week program of the intervention and were randomized to 1 of 3 different text message–based incentive conditions (gain-framed, loss-framed, or gain–social support).

**Main Outcomes and Measures:**

The primary outcome was change in anxious symptoms from baseline to end of treatment, as measured by the Hamilton Anxiety Rating Scale (HAM-A). The Anxiety Sensitivity Index and the Leibowitz Social Anxiety Scale scores were secondary measures.

**Results:**

The sample consisted of 59 participants (mean [SD] age, 23.1 [1.9] years; 46 [78%] female; 22 [37%] Asian, 3 [5%] Black, 5 [8%] Hispanic or Latino, 1 [2%] American Indian or Alaska Native, 25 [42%] White, and 6 [10%] >1 race; 32 [54%] college-educated and 12 [20%] graduate or professional school-educated; mean [SD] baseline HAM-A score, 15.0 [6.5]). Anxiety, measured by HAM-A, decreased across conditions from baseline to end of the intervention (mean difference, −5.64; 95% CI, −7.23 to −4.05), and symptomatic improvement was maintained at the week 12 follow-up (baseline to follow-up mean difference, −5.67; 95% CI, −7.29 to −4.04). However, there was no evidence that change in anxiety differed by incentive condition (loss-framed vs gain–social support mean difference, −1.40; 95% CI, −4.72 to 1.93; gain-framed vs gain–social support mean difference, 1.38; 95% CI, −1.19 to 3.96). Secondary anxiety measures (Anxiety Sensitivity Index and Liebowitz Social Anxiety Scale scores) showed a similar pattern of improvement, with no evidence of differences between incentive conditions. Participants completed most of the 12 sessions (mean [SD], 10.8 [2.1]; 95% CI, 10.3-11.4), and User Mobile Application Rating Scale app quality ratings exceeded the published threshold for acceptability at all study visits. There was no evidence that either session completion or app quality ratings differed by incentive condition.

**Conclusions and Relevance:**

In this randomized clinical trial of an app-based intervention for anxiety, the primary hypothesis that improvement in anxiety would be greatest in the condition using gain of points plus social incentives was not supported; however, the results suggest that a CBT application incorporating a full suite of CBT skills and embedded user engagement features was efficacious in improving symptoms in young adults with anxiety disorders. Given these findings, digital interventions represent a promising step toward wider dissemination of high-quality, evidence-based interventions.

**Trial Registration:**

ClinicalTrials.gov Identifier: NCT05130281

## Key Points

**Question:**

Does the efficacy of a self-guided cognitive behavioral therapy mobile app for young adults with anxiety disorders, differ by incentive conditions used to promote user engagement?

**Findings:**

In this randomized clinical trial of 59 young adults, change in anxiety from baseline to end of the intervention did not differ significantly between groups incentivized by loss of points, gain of points, or gain of points plus social support. However, there was a large and significant decrease in anxiety across incentive conditions.

**Meaning:**

The findings of this study suggest that self-guided mobile cognitive behavioral therapy apps can be efficacious in improving anxiety symptoms, regardless of the type of incentive strategy used to promote engagement.

## Teaser

This randomized clinical trial examines the efficacy of a self-guided mobile cognitive behavioral therapy intervention among young adults with anxiety disorders, comparing the effects of 3 different incentive conditions.

## Introduction

Anxiety disorders have been increasing among 18- to 25-year-old individuals more rapidly than in any other adult age range, with recent past-month prevalence estimates reaching 15%.^1,2^ A US Surgeon General advisory^3^ emphasizes that although the rate of change accelerated during the COVID-19 pandemic, psychiatric diagnoses among young people were already increasing before the pandemic. Anxiety disorders are associated with poor quality of life, difficulty working, disruption in social relationships and academic achievement, and financial burden.^4,5,6,7,8,9,10^

These disorders can be treated effectively by psychotherapeutic interventions, and a majority of patients, particularly young adults, express a preference for psychotherapy over psychotropic medication.^11^ Cognitive behavioral therapy (CBT) is considered a gold standard for treatment of anxiety^12^ on the basis of abundant evidence of efficacy.^13,14^ However, despite the existence of efficacious interventions, more than 25% of young adults perceive an unmet need for mental health treatment,^15,16^ in part because of challenges in accessing such treatment. Reluctance to discuss mental health problems, financial constraints, geographic distance from mental health services, and limited clinician training in CBT approaches all represent barriers to accessing care.^3,16,17,18^

Digital interventions, such as smartphone applications, allow treatment at home, are more affordable than paying for ongoing traditional mental health care, and do not rely on the ability to access a trained, local therapist. The increased prevalence of anxiety has not been met by an increased supply of mental health professionals, and digital access to evidence-based, self-guided CBT represents a scalable alternative. Self-guided CBT apps have demonstrated efficacy in adults with depression,^19,20,21^ but few studies have investigated their efficacy in individuals with anxiety disorders.^22,23^

Self-guided mental health apps have been stymied by 2 limitations. First, most self-guided apps provide an incomplete representation of the psychotherapies on which they are based. In particular, they do not emphasize behavioral strategies (eg, exposure to anxiety-provoking situations),^24,25^ a key therapeutic component of interventions for anxiety.^26,27^ Second, low user engagement diminishes their potential impact.^28,29^ Engagement may be improved by involving social support in the intervention^29^ or by incorporating behavioral economics and gamification.^30,31^

The Maya app is a digital CBT intervention targeting anxiety in young adults that addresses the limitations of existing apps. The app teaches a comprehensive array of CBT skills that target common features of anxiety, with a focus on behavioral skills often omitted from other apps. The app uses a visually appealing user interface and incorporates engagement strategies drawn from behavioral economics.^30,31,32,33,34^ Material is presented in an interactive format to encourage active participation, which leads to better learning and greater engagement than passive viewing of information.^35^ In addition to these features, the app can be used in conjunction with a text-based, gamified incentive system to further encourage engagement.

The primary aim of the current study was to compare the effects of 3 incentive strategies on the efficacy of the app in improving anxiety in adults aged 18 to 25 years with anxiety disorders. Given the powerful impact of social feedback in adolescence and early adulthood,^36,37,38^ our primary hypothesis was that anxiety would decrease more for participants incentivized by receipt of reward points combined with social support than for participants incentivized by either gain or loss of reward points alone.^29,39,40^ The secondary aims of the study were to evaluate engagement and self-reported satisfaction with the app and to compare these results among the incentive conditions.

## Methods

### Study Design

This randomized clinical trial examined improvement of anxiety during the intervention accompanied by 1 of 3 incentive conditions to promote engagement. In the absence of sufficient preliminary data to support a formal power calculation, an intended sample size of 120 was determined based on prior investigations of similar design (ie, randomized clinical trials of self-guided smartphone app interventions for anxiety or depression).^22,41,42,43^ However, the full sample was not recruited due to disruption of research procedures during the COVID-19 pandemic. Participants were individuals aged 18 to 25 years with anxiety disorders, who were assigned to use the app for 6 weeks and were randomized to a loss-framed, gain-framed, or gain–social support incentivization. Anxiety was assessed at baseline, week 3 (midpoint), week 6 (end of intervention), and week 12 (follow-up). Change in anxiety (Hamilton Anxiety Rating Scale [HAM-A]^44^) at week 6 (end of intervention) was the primary end point. Approval for the trial protocol (Supplement 1) was obtained from the Weill Cornell Medicine Institutional Review Board before study initiation. Written consent was obtained remotely using an electronic version of the informed consent form that followed federal, state, and local regulations, as applicable. This report follows the Consolidated Standards of Reporting Trials (CONSORT) reporting guideline for randomized clinical trials.

### Participants

**Figure 1.**
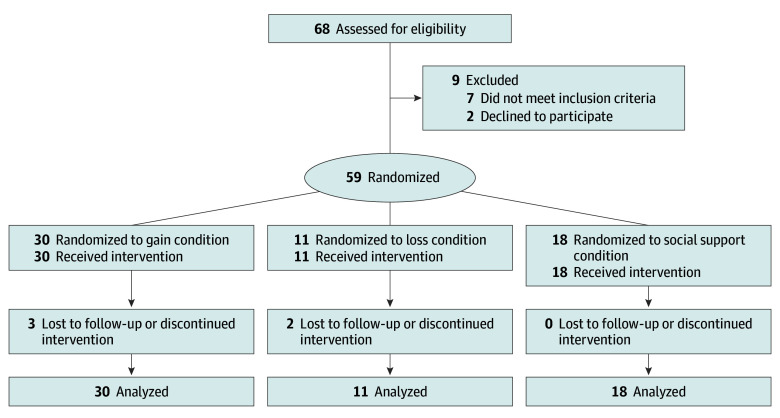
CONSORT Diagram Illustrating Number of Participants Included at Each Point in the Study From Enrollment to Analysis

Participants were recruited through online advertisements and the outpatient psychiatry clinics of Weill Cornell Medicine. Eligible participants had a primary anxiety disorder diagnosis, determined by a score of 4 or higher on the clinical severity rating of the Anxiety and Related Disorders Interview Schedule for *DSM-5* (ADIS-5)^45^ and were fluent in English. Race and ethnicity were self-reported by participants from categories defined by investigators to provide information about the representativeness of the sample. Participants were excluded if they were already participating in CBT, had changed psychotropic medication dosage in the past 12 weeks, or endorsed suicidal ideation with intent and/or plan (Figure 1).

### Study Intervention

The content of the app was developed by clinicians and researchers at Weill Cornell Medicine, and the app is free to download. The current publicly available version of the app includes the same core skills as the version used for the current study but includes language with a greater focus on wellness and resilience, has fewer required exercises, and allows for greater user choice of activities. Participants were assigned to complete 12 sessions of the intervention during 6 weeks (2 sessions per week). Modules included psychoeducational videos and quizzes, cognitive restructuring exercises, exposure exercises, mindfulness, and additional CBT skills (eFigure in Supplement 2). Each session included homework to be completed before the next session.

### Incentive Conditions

The incentive conditions consisted of the following: (1) a loss-framed condition in which participants began each week with a specified number of points that they could lose for not completing assigned sessions on time; (2) a gain-framed condition in which participants could earn points for completing assigned sessions; and (3) a gain–social support condition that included all aspects of the gain-framed condition plus the ability to designate a friend or family member to receive text updates about the participant’s progress and provide social support. Participants received virtual “medals” (bronze, silver, gold, or platinum) for obtaining specified levels of points. If a participant in the gain–social support condition declined to designate a friend or family member, a research assistant not involved in collection of study data was designated as the social support person. The content and format of the app sessions did not differ among the incentive conditions.

Incentives were delivered separately from the app via text messages sent through Way to Health,^46^ a research platform developed by the University of Pennsylvania Center for Health Incentives and Behavioral Economics.^47,48,49^ Text messages included reminders, gamification incentives (points and badges), and, where relevant, updates to the social support person with suggested messages of support (eg, “[Participant] has completed the third week of the program! Let me tell you about some achievements. [Participant] earned 1800 points for completing sessions on time. Encourage [Participant] to keep this up.”).

### Randomization

Randomization was conducted through the Way to Health platform, which generated the random allocation sequence. Participants were randomized to 1 of 3 incentive conditions according to a simple 1:1:1 nonblocked design through the Way to Health platform. Study research assistants (M.M.S.) enrolled participants and were masked to the randomization assignment. Because targeted enrollment was not achieved due to the COVID-19 pandemic and the 1:1:1 randomization scheme did not specify block randomization, participants were allocated unevenly to the 3 conditions.

### Modifications Due to the COVID-19 Pandemic

This trial was intended to begin in March 2020, but study initiation was delayed until June 2021 after nonessential research activities at Weill Cornell Medicine were paused due to the COVID-19 pandemic. Therefore, enrollment was between June 16, 2021, and November 11, 2022. Our recruitment target of 120 participants was amended as a result, which reduced power and led to imbalanced comparison groups. The trial concluded when the end of the preplanned study period was reached. We report methods and results in accordance with guidelines for clinical trials affected by the pandemic.^50^

### Measures

The ADIS-5^45^ was administered to determine psychiatric diagnosis and clinical severity rating at baseline and end point. The HAM-A^44^ score was the primary measure of anxiety. The Anxiety Sensitivity Index (ASI)^51^ and Liebowitz Social Anxiety Scale (LSAS)^52^ scores were secondary measures of anxiety. The 24-item Hamilton Depression Rating Scale^53^ was the measure of depressive symptoms (exploratory efficacy outcome). App engagement was measured in terms of the total number of sessions completed. Retention was assessed at week 6 (end of intervention) and week 12 (follow-up). Participant satisfaction was measured with the User Version of the Mobile Application Rating Scale (uMARS).^54^ Detailed descriptions of measures and training procedures are provided in the eMethods in Supplement 2.

### Procedure

Study visits were conducted remotely via a Health Insurance Portability and Accountability Act–compliant videoconferencing platform (Zoom, versions 5.6.7-5.12.9; Zoom Video Communications Inc). The baseline assessment included the measures described above and a session to assist the participant with app setup. App programs were set to begin the Sunday after enrollment. Outcome measures and qualitative feedback questionnaires about the app were collected at weeks 3 (midpoint), 6 (end of intervention), and 12 (follow-up). The uMARS was assessed beginning at week 1 rather than at baseline to allow participants to use the app before rating it. The week 3 and 6 visits also included troubleshooting of technical difficulties.

### Statistical Analysis

Statistical analyses were conducted with Jamovi, version 2.3.2.0,^55^ a software package based on the R statistical language (R Foundation for Statistical Computing). To evaluate the effect of the intervention on primary (HAM-A) and secondary (ASI and LSAS) measures of anxiety, we used linear mixed-effects models with subject intercept as a random effect. All participants with baseline data were included following an intent-to-treat approach. Linear mixed-effects models used the restricted maximum likelihood estimation method, which accounts for missing values without removing entire participants from the analysis. We tested the main effect of time (baseline [week 0], midpoint [week 3], end point [week 6], and follow-up [week 12]) as a fixed effect, incentive condition (gain–social support, gain-framed, and loss-framed) as a fixed effect, and a time × incentive condition interaction.

To compare participant engagement (sessions completed) among the incentive conditions, because of the imbalance in sample size across conditions, we used a nonparametric Kruskal-Wallis test. To evaluate participant satisfaction with the intervention, we computed a linear mixed-effects model with a random effect of participants, fixed effects of time (week 1, midpoint, end point, and follow-up) and incentive condition (gain–social support, gain-framed, and loss-framed), a time × incentive condition interaction, and the total uMARS App Quality score as the outcome. For descriptive purposes, we calculated the percentage of the sample who rated the app quality as a 4 or greater on the uMARS, based on the published threshold for app satisfaction.

Post hoc 2-tailed, paired *t* test analyses of change in assessment scores from baseline to midpoint, end point, and follow-up used the Holm method to correct for multiple comparisons. Effect sizes are reported in terms of Cohen *d*, in which *d* = 0.2 represents a small effect, *d* = 0.5 represents a medium effect, and *d* = 0.8 represents a large effect.^56^ A *P* value threshold of .05 was used to determine significance. Data analysis was performed from December 21, 2022, to June 14, 2024. Exploratory analyses are reported in the eMethods in Supplement 2.

## Results

### Sample Characteristics

Sixty-eight prospective participants were assessed, and 59 (mean [SD] age, 23.1 [1.9] years; 46 [78%] female and 13 [22%] male; 22 [37%] Asian, 3 [5%] Black, 5 [8%] Hispanic or Latino, 1 [2%] American Indian or Alaska Native, 25 [42%] White, and 6 [10%] >1 race; 32 [54%] college-educated and 12 [20%] graduate or professional school–educated) were enrolled and randomized (Figure 1). Participants presented most frequently with generalized anxiety disorder (33 [56%]) followed by social anxiety disorder (24 [41%]). The mean (SD) baseline HAM-A score was 15.0 (6.5). Demographic and clinical characteristics of the sample are provided in Table 1. No participants reported serious adverse events.

**Table 1.** Sample Characteristics^a^

Characteristic	Full sample (N = 59)	Gain framed (n = 30)	Loss framed (n = 11)	Social support (n = 18)
Age, mean (SD), y	23.1 (1.9)	22.9 (1.9)	22.4 (2.4)	24.0 (1.2)
Gender				
Female	46 (78)	23 (77)	5 (45)	18 (100)
Male	11 (19)	7 (20)	4 (36)	0
Nonbinary	1 (2)	0	1 (9)	0
Did not answer	1 (2)	0	1 (9)	0
Educational level				
Graduate/ or professional	12 (20)	7 (23)	1 (9)	4 (22)
College	32 (54)	16 (53)	4 (36)	12 (67)
Some college	4 (7)	1 (3)	2 (18)	1 (6)
Associate degree	0	0	0	0
High school	6 (10)	4 (13)	2 (18)	0
Did not answer	5 (8)	2 (7)	2 (18)	1 (6)
Race				
Asian	22 (38)	14 (47)	2 (18)	6 (33)
Black	3 (5)	0	0	3 (17)
American Indian or Alaska Native	1 (1)	0	0	1 (6)
White	26 (44)	13 (43)	6 (55)	7 (39)
>1 Race	3 (5)	3 (10)	0	0
Did not answer	1 (2)	0	1 (9)	0
Other, not specified	3 (3)	0	0	1 (9)
Ethnicity				
Hispanic or Latino	5 (8)	1 (3)	3 (27)	1 (6)
Non-Hispanic or Latino	52 (88)	28 (93)	7 (64)	17 (94)
Other (did not answer)	2 (3)	1 (3)	1 (9)	0
Principal diagnosis^b^				
Generalized anxiety disorder	33 (56)	16 (53)	6 (55)	11(61)
Social anxiety disorder	24 (41)	12 (40)	4 (36)	8 (44)
Panic disorder	4 (7)	3 (10)	0	1 (6)
Agoraphobia	1 (2)	0	1 (9)	0
Specific phobia	1 (2)	1 (3)	0	0
Other specified	1 (2)	0	1 (9)	0
Clinical severity rating, mean (SD)^c^				
Baseline	5.2 (0.8)	5.4 (0.8)	5 (0.8)	5.1 (0.7)
End point	3.7 (1.3)	4.1 (1.2)	3.2 (1.3)	3.4 (1.1)
Comorbid diagnoses^d^				
Anxiety disorders	41 (70)	21 (70)	7 (64)	13 (72)
Depressive disorders	12 (20)	6 (20)	2 (18)	4 (22)
Obsessive-compulsive and related disorders	4 (7)	1 (3)	2 (18)	1 (6)
Attention-deficit/hyperactivity disorder				
Substance use disorder	1 (2)	1 (3)	0	0
Baseline anxiety score (HAM-A), mean (SD)	15.0 (6.5)	16.7 (7.3)	11.5 (3.4)	14.3 (5.8)
Baseline anxiety sensitivity score (ASI), mean (SD)	25.3 (14.2)	28.1 (15.1)	22.3 (12.4)	22.4 (13.3)
Baseline social anxiety score (LSAS), mean (SD)	52.7 (27.8)	59.3 (26.8)	44.2 (29.7)	47.1 (27.3)
Baseline depression score (HAM-D), mean (SD)	15.9 (5.9)	16.4 (6.2)	14.4 (4.3)	15.9 (6.3)

Abbreviations: ASI, Anxiety Sensitivity Index; HAM-A, Hamilton Anxiety Rating Scale; HAM-D, Hamilton Depression Rating Scale; LSAS, Liebowitz Social Anxiety Scale.

^a^ Data are presented as number (percentage) of participants unless otherwise indicated.

^b^ Frequency counts for principal diagnoses exceed the sample size because if an individual had 2 co-occurring anxiety disorders at the same clinical severity rating, both were counted. Uneven group sizes reflect lack of block randomization; see text for details.

^c^ Clinical severity rating is a component of the Anxiety and Related Disorders Interview Schedule for *DSM-5*, determined by the clinical interviewer according to a set of anchor points from 0 to 8 based on severity of reported symptoms.

^d^ Although all participants had a principal diagnosis of an anxiety disorder, anxiety disorders were considered a comorbidity if the participant had a second anxiety disorder in addition to the principal diagnosis.

### Efficacy on Anxiety

**Figure 2.**
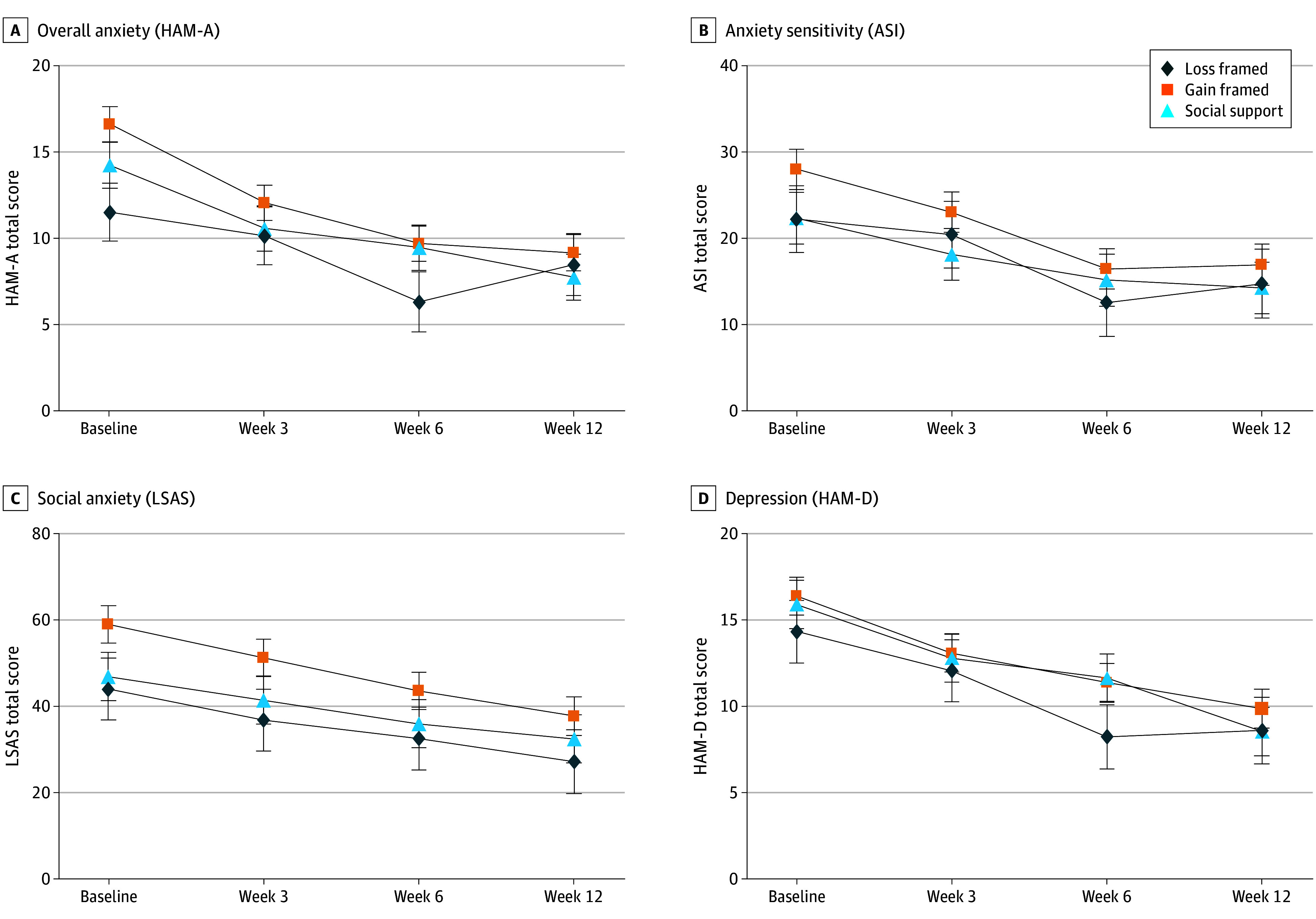
Results of the Linear Mixed-Effects Model Demonstrating Change in Outcome Measure by Time and Incentive Condition Error bars represent SEs. ASI indicates Anxiety Sensitivity Index; HAM-A, Hamilton Anxiety Rating Scale; HAM-D, Hamilton Depression Rating Scale; and LSAS, Liebowitz Social Anxiety Scale.

There was a significant main effect of time on the primary measure of anxiety (HAM-A scores): anxiety was significantly lower at midpoint (week 3 mean difference, −3.20; 95% CI, −4.76 to −1.64; Cohen *d* = 0.64), end point (week 6 mean difference, −5.64; 95% CI, −7.23 to −4.05; Cohen *d* = 0.94), and follow-up (week 12 mean difference, −5.67; 95% CI, −7.29 to −4.04; Cohen *d* = 1.04) than baseline (Figure 2). Effect sizes were large at end point and follow-up. There was no evidence that change in anxiety differed by incentive condition (loss-framed vs gain–social support mean difference, −1.40; 95% CI, −4.72 to 1.93; gain-framed vs gain–social support mean difference, 1.38; 95% CI, −1.19 to 3.96) or time × incentive condition interaction (loss-framed and gain–social support mean difference, 2.30; 95% CI, −1.92 to 6.53 at midpoint; −0.43; 95% CI, −4.74 to 3.88 at end point; and 3.47; 95% CI, −0.94 to 7.88 at follow-up; gain-framed and gain–social support mean difference, −0.90; 95% CI, −4.19 to 2.39 at midpoint; −2.16; 95% CI, −5.45 to 1.14 at end point; and −0.97; 95% CI, −4.30 to 2.37 at follow-up) (Table 2).

**Table 2.** Results of Linear Mixed Models for the Main Effect of Time Demonstrating Estimates of Mean Change by Time

Parameter estimate	Estimate (95% CI)	*t*	*P* value
**HAM-A**			
Intercept	10.54 (9.31 to 11.11)	16.82	<.001
Midpoint-baseline	−3.20 (−4.76 to −1.64)	−4.02	<.001
End point–baseline	−5.64 (−7.23 to −4.05)	−6.97	<.001
Follow-up–baseline	−5.67 (−7.29 to −4.04)	−6.83	<.001
Loss framed and gain social	−1.40 (−4.72 to 1.93)	−0.82	.41
Gain framed and gain social	1.38 (−1.19 to 3.96)	1.05	.30
Midpoint-baseline × loss framed and gain social	2.30 (−1.92 to 6.53)	1.07	.29
End point–baseline × loss framed and gain social	−0.43 (−4.74 to 3.88)	−0.20	.85
Follow-up–baseline × loss framed and gain social	3.47 (−0.94 to 7.88)	1.54	.13
Midpoint-baseline × gain framed and gain social	−0.90 (−4.19 to 2.39)	−0.54	.59
End point–baseline × gain framed and gain social	−2.16 (−5.45 to 1.14)	−1.28	.20
Follow-up–baseline × gain framed and gain social	−0.97 (−4.30 to 2.37)	−0.57	.57
**ASI**			
Intercept	18.73 (15.52 to 21.94)	11.44	<.001
Midpoint-baseline	−3.69 (−6.22 to −1.17)	−2.87	.005
End point–baseline	−9.51 (−12.08 to −6.94)	−7.26	<.001
Follow up–baseline	−8.90 (−11.54 to −6.27)	−6.62	<.001
Loss framed and gain social	0.03 (−8.66 to 8.72)	0.01	.99
Gain framed and gain social	3.65 (−3.10 to 10.41)	1.06	.29
Midpoint-baseline × loss framed and gain social	2.40 (−4.43 to 9.24)	0.69	.49
End point–baseline × loss framed and gain social	−2.46 (−9.44 to 4.53)	−0.69	.49
Follow-up–baseline × loss framed and gain social	0.63 (−6.51 to 7.78)	0.17	.86
Midpoint-baseline × gain framed and gain social	−0.81 (−6.14 to 4.51)	−0.30	.77
End point–baseline × gain framed and gain social	−4.41 (−9.74 to 0.91)	−1.62	.11
Follow-up–baseline × gain framed and gain social	−3.00 (−8.40 to 2.39)	1.09	.28
**LSAS**			
Intercept	40.91 (34.92 to 46.90)	13.39	<.001
Midpoint-baseline	−6.84 (−11.46 to −2.21)	−2.90	.004
End point–baseline	−12.67 (−17.38 to −7.96)	−5.27	<.001
Follow-up–baseline	−17.61 (−22.45 to −12.77)	−7.13	<.001
Loss framed and gain social	−4.01 (−20.23 to 12.21)	−0.48	.63
Gain framed and gain social	8.78 (−3.83 to 21.38)	1.36	0.18
Midpoint-baseline × loss framed and gain social	−1.63 (−14.15 to 10.90)	−0.25	.80
End point–baseline × loss framed and gain social	−0.40 (−13.20 to 12.41)	−0.06	.95
Follow-up–baseline × loss framed and gain social	−2.30 (−15.40 to 10.80)	−0.34	.73
Midpoint-baseline × gain framed and gain social	−2.21 (−11.97 to 7.55)	−0.44	.66
End point–baseline × gain framed and gain social	−4.44 (−14.20 to 5.31)	−0.89	.37
Follow-up–baseline × gain framed and gain social	−6.86 (−16.80 to 3.08)	−1.35	.18

Abbreviations: ASI, Anxiety Sensitivity Index; HAM-A, Hamilton Anxiety Rating Scale; LSAS, Liebowitz Social Anxiety Scale.

For the secondary measures of anxiety, there was a significant main effect of time on the ASI and the LSAS. There were significant decreases in severity of anxiety sensitivity at midpoint (week 3 mean difference, −3.69; 95% CI, −6.22 to −1.17; Cohen *d* = 0.49), end point (week 6 mean difference, −9.51; 95% CI, −12.08 to −6.94; Cohen *d* = 0.93), and follow-up (week 12 mean difference, −8.90; 95% CI, −11.54 to −6.27; Cohen *d* = 0.93) (Figure 2). There were also significant decreases in severity of social anxiety symptoms at midpoint (week 3 mean difference, −6.84; 95% CI, −11.46 to −2.21; Cohen *d* = 0.47), end point (week 6 mean difference, −12.67; 95% CI, −17.38 to −7.96; Cohen *d* = 0.70), and follow-up (week 12 mean difference, −17.61; 95% CI, −22.45 to −12.77; Cohen *d* = 1.07) (Figure 2). Effect sizes were moderate to large. There were no statistically significant main effects of incentive or time × incentive condition interactions for the ASI or LSAS (Table 2).

### Engagement

Overall, 58 of the 59 participants (98%) completed the week 6 end point assessment, and 55 of 59 (93%) completed the subsequent week 12 follow-up. On average, participants completed most of the 12 intervention sessions (mean [SD], 10.8 [2.1]; 95% CI, 10.3-11.4). Thirty-eight of 59 participants (64%) completed all sessions in the program. Of those who completed the follow-up assessment, 21 of 55 (39%) reported that they continued to use the app beyond the 6-week timeframe. The number of sessions completed did not differ significantly by participant age (Pearson *r* = 0.03, *P* = .81), gender (male vs female mean difference, −0.82; 95% CI, −2.43 to 0.79; nonbinary gender was not included in analysis because only 1 individual so identified), or race (White vs races other than White mean difference, −0.47; 95% CI, −1.60 to 0.65). There was no evidence that number of sessions completed differed by incentive condition (gain-framed vs loss-framed mean difference, −0.67; 95% CI, −2.75 to 1.42; gain–social support vs loss-framed mean difference, −1.56; 95% CI, −3.55 to 0.44; gain-framed vs gain–social support mean difference, −0.89; 95% CI, −1.82 to 0.04).

### Participant Satisfaction

A linear mixed-effects model of the uMARS App Quality score indicated no significant fixed effect of time (week 1 vs midpoint mean difference, 0.07; 95% CI, −0.06 to 0.2; week 1 vs end point mean difference, 0.07; 95% CI, −0.07 to 0.2; week 1 vs follow-up mean difference, 0.14; 95% CI, 0.0002-0.27) or incentive condition (loss-framed vs gain–social support mean difference, −0.16; 95% CI, −0.49 to 0.17; gain-framed vs gain–social support mean difference, −0.01; 95% CI, −0.26 to 0.25) and no significant time × incentive group interaction. At each time point and regardless of incentive group, the mean uMARS App Quality score was greater than the published cutoff for acceptable app satisfaction of 4 (week 1 mean, 4.09; 95% CI, 3.96-4.23; 39 of 57 participants [68%] with scores >4; week 3 mean, 4.17; 95% CI, 4.04-4.30; 40 of 59 participants [68%] with scores >4; end point mean, 4.18; 95% CI, 4.05-4.32; 41 of 58 participants [71%] with scores >4; and follow-up mean, 4.22; 95% CI, 4.09-4.36; 35 of 52 participants [68%] with scores >4). Additional linear mixed-model results are given in the eResults in Supplement 2.

## Discussion

Contrary to our primary hypothesis, we did not find evidence of a difference in efficacy among the 3 incentive conditions. However, after 6 weeks of use of the app, there was a clinically and statistically significant decrease in anxiety symptoms across conditions. This decrease in anxiety persisted at the week 12 follow-up. Study retention, app engagement, and participant satisfaction with the app were high across incentive conditions and did not differ by condition. All but 1 participant remained in the study during the intervention period, and a majority completed all intervention sessions. At least 68% of participants assigned a mean app quality rating greater than 4 of 5 at each time point.

The small size of comparator groups may have limited our ability to detect group differences by incentive condition. The absence of a group difference may also reflect a ceiling effect on the engagement and efficacy of the app. In addition to the incentive strategies used in conjunction with the app, the app itself includes numerous features intended to enhance both engagement and efficacy: a visually appealing user interface, a progress monitoring feature, interactive modules with activities designed to maintain users’ attention, and comprehensive coverage of CBT and adjunctive skills. Moreover, all 3 incentive conditions involved either a gain-framed or a loss-framed component, both of which are efficacious in changing health-related behaviors.^40^

Effect sizes of the decrease in anxiety across conditions were moderate to large, suggesting a clinically significant association between use of the intervention and improvement of anxiety that was greater than those typically observed in digital mental health interventions^31,57,58^ and on par with pharmacologic trials.^59,60,61^ Secondary analyses indicated that anxiety remained lower than baseline at a week 12 follow-up assessment (6 weeks after the end of the intervention) and did not differ between the end of the intervention and follow-up, suggesting potential persistence of benefit. This finding is consistent with prior work that found sustained effects of traditional therapist-led CBT after treatment termination.^62,63^

Similar to overall anxiety, both anxiety sensitivity (fear of fear) and social anxiety decreased from baseline to the end of the intervention, and improvement persisted at follow-up. Our findings suggest that, as intended, the intervention may target specific anxiety symptom clusters in addition to overall anxiety. It remains to be determined whether individuals with clinical profiles characterized by anxiety sensitivity, social anxiety, or other symptoms would respond equally well to a more streamlined app that includes only the most relevant modules.

We measured high levels of engagement and participant satisfaction with the app across incentive conditions, supporting the feasibility of the app for young adults with anxiety. There was a 98% retention rate compared with the 50% retention reported for other randomized clinical trials of mental health apps,^64^ and 64% of participants completed all required sessions compared with the 35% reported in the literature.^65^ The app could be scaled up for wider use because it does not require significant financial resources on the part of the user or a clinician. It is free to download and uses incentive systems based on points rather than monetary incentives. Additional discussion points are given in the eDiscussion in Supplement 2.

### Limitations

This study had several limitations. Because the full sample could not be recruited as intended and we did not use block randomization, participants were not allocated evenly to the 3 incentive groups. The unbalanced allocation and reduced sample size decrease our capacity to detect group differences.^66^ Another limitation was the absence of a control condition in which participants did not receive the app or received the app alone without additional incentives. We elected not to include such a control group to allow for the inclusion of 3 incentive conditions within the constraints of the available resources. However, this study design does not allow us to estimate the extent to which symptom changes reflect regression to the mean or placebo-like effects. That said, although untreated individuals show spontaneous improvement of anxiety, the effect size of this improvement is typically small.^67^ It is therefore unlikely that the moderate to large reductions of anxiety observed in the current study were spurious. Finally, the sample was predominantly female, college educated, and of Asian or White race and non-Hispanic ethnicity, limiting conclusions about generalizability to more diverse groups.

## Conclusions

The results of this study demonstrate the efficacy and feasibility of a comprehensive, scalable, self-guided mobile CBT intervention for young adults with anxiety, which did not differ significantly by incentive condition. The Maya app includes design features, an engaging user interface, and broad coverage of CBT techniques that address the limitations of existing digital mental health apps. In the context of a mental health crisis in young adults and numerous barriers to traditional mental health services, digital interventions represent a promising step toward wider dissemination of high-quality, evidence-based interventions. Such interventions have the potential to help with shortages of mental health professionals and long wait times and to address geographic and socioeconomic disparities in access to care. Treatment programs that incorporate apps may be a viable strategy to address the persistent lack of access to adequate mental health care^3^ that exists even in resource-rich settings.

## Notes

Supplement 1.
Trial Protocol

Supplement 2. **eFigure.** Therapeutic Themes, Symptom Targets, and Modules Included at Each Week of the Maya Program **eMethods.** Descriptions of Measures and Training Procedures **eResults.** Linear Mixed Model Results by Outcome **eDiscussion.** Supplementary Discussion
eReferences

Supplement 3.
Data Sharing Statement
