# Supplementary material for: Efficacy of a Mobile App-Based Intervention for Young Adults With Anxiety Disorders: A Randomized Clinical Trial
Source: JAMA Netw Open. 2024 Aug 20;7(8):e2428372. doi: 10.1001/jamanetworkopen.2024.28372 (PMC11337073; doi:10.1001/jamanetworkopen.2024.28372)
Supplement: Supplement 1. — Trial Protocol [file jamanetwopen-e2428372-s001.pdf]

TITLE: Mobile Cognitive Behavior Therapy Targeting Anxiety Disorders

Version Date: 5/01/2021

Principal Investigator: Faith Gunning
Weill Cornell Medicine
212-746-4666
fgd2002@med.cornell.edu

Participating Sites: *Sackler Institute, WCM. The PI is Faith Gunning. See above for contact info.*

List of Abbreviations

**AE**	Adverse Event
**CFR**	Code of Federal Regulations
**CRF**	Case Report Form
**CTSC**	Clinical Translational Science Center
**DSMB**	Data Safety Monitoring Board
**DSMP**	Data Safety Monitoring Plan
**FDA**	Food and Drug Administration
**GCP**	Good Clinical Practice
**HIPAA**	Health Insurance Portability and Accountability Act of 1996
**HRBFA**	Human Research Billing Analysis Form
**HUD**	Humanitarian Use Device
**ICF**	Informed Consent Form
**IDE**	Investigational Device Exemption
**IND**	Investigational New Drug
**IRB**	Institutional Review Board
**PHI**	Protected Health Information
**PI**	Principal Investigator
**REDCap**	Research Electronic Data Capture
**SAE**	Serious Adverse Event
**SUSAR**	Suspected Unexpected Serious Adverse Reaction
**UAP**	Unanticipated Problem
**WCM**	Weill Cornell Medicine

Protocol Summary

**Full Title:**	Mobile Cognitive Behavior Therapy Targeting Anxiety Disorders
**Short Title:**	Mobile CBT in Anxiety
**Clinical Phase:**	N/A – no drug or device involved
**Principal Investigator:**	Faith Gunning
**Sample Size:**	N = 120
**Accrual Ceiling:**	We plan to screen up to 300 subjects.
**Study Population:**	18-25 year olds who have been diagnosed with an anxiety disorder as per the Anxiety Disorders Interview Schedule.
**Accrual Period:**	We anticipate complete accrual of participants taking up to 1 year
**Study Design:**	Participants in this study will use an online Cognitive Behavior Therapy program to assist with treatment of their anxiety symptoms. This program is being developed by Weill Cornell Medicine faculty as part of the University of Pennsylvania Center for Health Incentives and Behavioral Economics's established online platform called "Way to Health." All participants in the study will be using the program; as this is a pilot study testing the feasibility of using an online CBT program plus non-monetary incentives. Participants will be told to use the program for at least 20 minutes per day, 2 days per week, for up to 6 weeks. Participants will check in once a week in person or over the phone or a HIPAA-compliant virtual meeting platform. There will also be one follow up visit 6 weeks after they have stopped using the program.
**Study Duration:**	3 months
**Study Agent/ Intervention Description:**	There is no drug or invasive device being used; rather, we are simply providing an online Cognitive Behavior Therapy program through an existing online platform called "Way to Health"
**Primary Objective:**	The primary objective is to examine different ways of motivating people to make use of the online therapy program, and to assess whether social supports are as efficacious, or more efficacious, than a reward mechanism such as non-monetary incentives. We will randomize subjects to one of three conditions: a social support condition, a gain-framed condition in which participants can earn "points" for completing their assigned sessions, and a

loss-framed condition in which participants lose “points” for failing to complete their assigned sessions on time. We will compare change in anxiety from baseline to end of treatment between these three conditions.

Secondary Objectives: A secondary objective will be to evaluate whether the three incentive conditions are associated with different levels of treatment engagement. We will measure the amount of time participants use the program and administer a questionnaire to assess participants’ subjective impressions of the program. We will compare these measures across the three incentive conditions.

Exploratory Objectives: We will collect neuroimaging data from a subset of participants who choose to participate in MRI scans and/or electroencephalographic (EEG) recordings. We will examine these scans for neural changes pre- to post-intervention. We will also assess associations between symptom improvement and time spent on different modules within the program.

Endpoints: We will administer several questionnaires that will assess severity of symptoms of anxiety in order to assess the efficacy of the program over time.

SCHEMA

All study assessments will be conducted either in person, over the phone, or on a HIPAA-compliant virtual meeting platform. Decision about how to conduct visits will be made with participants, depending on safety and convenience.

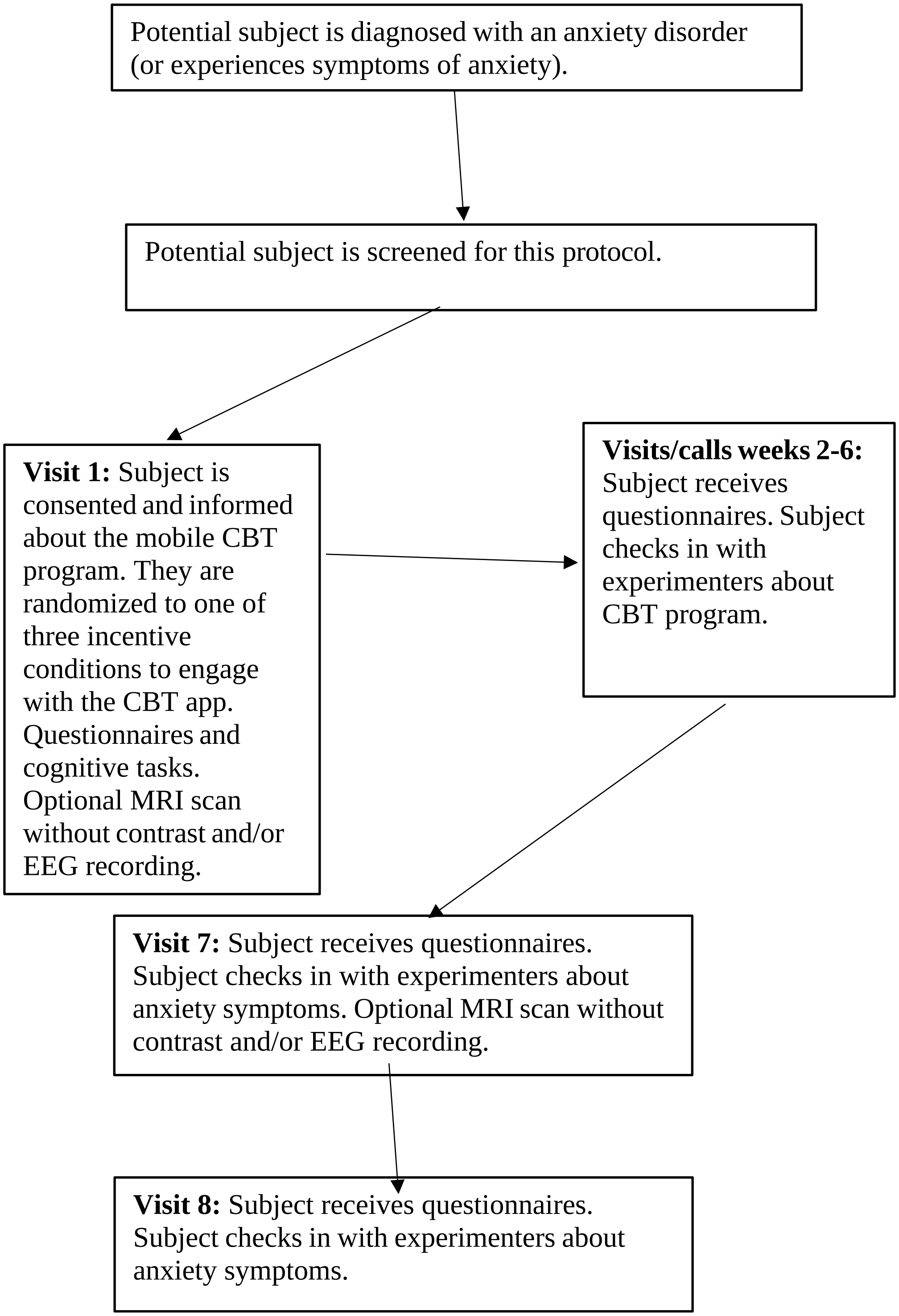

Table of Contents

List of Abbreviations 3

Protocol Summary 4

Study Agent/ 4

SCHEMA 3

Table of Contents 4

1. Study Objectives 6

1.1 Primary Objectives 6

1.2 Secondary Objectives 6

1.3 Exploratory Objectives 7

2. Background 7

2.1 Disease 7

2.2 Investigational Agent or Device 7

2.3 Rationale ..... **Error! Bookmark not defined.**

2.4 Risk/Benefit Assessment ..... **Error! Bookmark not defined.**

3. Subject Selection 8

3.1 Study Population 8

3.2 Inclusion Criteria 8

3.3 Exclusion Criteria 8

4. Registration Procedures 8

4.1 Patient Registration 9

5. Study Procedures 9

5.1 Schedule of Evaluations........ 9

5.2 Treatment Administration........ 12

5.3 General Concomitant Medication and Supportive Care Guidelines........ 12

5.4 Duration of Therapy and Criteria for Removal from Study........ 13

5.5 Duration of Follow Up........ 13

6. Measurement of Effect........ 13

7. Statistical Considerations........ 13

7.1 Study Design/Endpoints........ 13

7.2 Sample Size/Accrual Rate........ 14

7.3 Stratification Factors........ 14

Analysis of Endpoints........ 14

7.3.1........ 14

7.3.2 Analysis of Primary Endpoints........ 14

7.3.3 Analysis of Secondary Endpoints........ 14

1. Study Objectives

1.1 Primary Objectives

We plan to develop an online cognitive behavioral therapy program, in collaboration with the Center for Health Incentives and Behavioral Economics (Wharton School) that can be integrated into their online platform, Way to Health (https://www.waytohealth.org) that has been developed over the past 10 years and automates many of the research functions necessary for conducting randomized controlled trials of behavior interventions and strategic telehealth programs in areas related to primary care medicine. The platform is an efficient, scalable, and low cost way to test behavioral interventions. To date, the platform has supported NIH-funded interventions that focus on medication adherence; weight loss; monitoring of blood sugar, blood pressure and weight; physical activity; smoking cessation; sleep and patient compliance.

One of the key features of the online platform (Way to Health) is its capacity to embed various incentive tools such as gamification and social support to the CBT intervention. In this context, these incentive components are novel to the delivery of any CBT treatment. A main hypothesis to be tested in this objective is whether the social support component will have a particular advantage to increase efficacy of CBT in this age group. This social support component will potentially extend the benefits of a group therapy approach, including connection with others, support, and encouragement. To test this hypothesis, we will randomize participants to one of three incentive structure conditions: a gain-framed incentive in which participants earn points for completing sessions, a loss-framed incentive in which they lose points for failing to complete sessions, and a social support incentive in which participants can identify a person they know to receive updates about their progress and support them throughout their time in the program. Participants will not be required to choose a person they know to receive updates about their use of the program; instead, they can choose to have a member of the study team serve as their social support if they are assigned to this condition.

1.2 Secondary Objectives

In addition to assessing efficacy of the three incentive strategies, a secondary objective is to evaluate the extent to which each strategy enhances engagement with the CBT intervention. Necessary to the real-world success of any technology-based health intervention is the frequency with which the user interacts with the technology and the user’s perception of how helpful and easy to use it is; even the most well-designed treatment will not help anyone if people never use it. We hypothesize that the social support incentive strategy may increase use and enjoyment of the program. We will use both objective data collected by the online CBT program and subjective ratings reported by study participants to evaluate differences in engagement between the gain-framed, loss-

framed, and social incentive conditions.

1.3 Exploratory Objectives

We will collect neuroimaging data from a subset of participants who choose to participate in MRI scans and/or EEG recording pre-intervention and post-intervention. We will examine these scans for neural changes along with examining questionnaires and interviews to assess changes in symptoms pre- and post-intervention. We will also assess associations between symptom improvement and time spent on different modules within the program.

2. Background

2.1 Disease

The disease being researched is anxiety disorder, a category of psychiatric disorders that includes phobias, social anxiety, and panic disorders. It is characterized by stress that is disproportionate to the potential consequence of an event, an inability to stop worrying, and general restlessness. It has a one-year prevalence of 10.6% and a lifetime prevalence of 16.6%. In addition, it has seen a rise in prevalence in younger populations in recent years (Remes, 2016).

2.2 Investigational Agent or Device

The online CBT program will be developed with the consultation of a behavioral economics researcher at the University of Pennsylvania's Wharton School (Center for Health Incentives and Behavioral Economics; https://chibe.upenn.edu). The program will be integrated into their established online platform (Way to Health; https://www.waytohealth.org) that includes various incentive tools. Our program will

include modules addressing, among others: psychoeducation, emotion monitoring tools, cognitive restructuring strategies, mindfulness practices, exposure hierarchy building and maintenance tools, an evolving list of weekly exposure goals, exposure exercise processing tool, and as-needed motivational enhancement and relapse prevention modules. Module content will be continually updated based on ongoing patient assessment. For example, the exposure hierarchy will be an interactive tool with evolving fear and avoidance ratings based on weekly exposure exercises accomplished and anxiety data collected. These data will then inform recommendations for weekly exposure tasks, including when to repeat exposures and when to move on to a more challenging task. The online CBT platform will include an interactive dashboard to provide the user with their program "stats" based on patient goals will be entered into the system; it will provide prescribed outcome measures for tracking progress toward

goals. Participants will be told that they must engage with the online CBT platform for at least 20-25 minutes per day, 2 days per week. They are allowed to use the program more frequently than this if they want to.

Although the proposed version of this app is new, the content and its structure are similar to how Cognitive Behavioral Therapy (CBT) is commonly delivered. We have a collaboration with University of Pennsylvania's Wharton School (Center for Health Incentives and Behavioral Economics; https://chibe.upenn.edu), who have an established online platform called Way to Health. Way to Health has been used to deliver interventions to thousands of patients. In 2017, the platform supported 38 research projects and 11 clinical pilots. We are working closely with Kevin Volpp, M.D., Ph.D. at University of Pennsylvania who has demonstrated how incentives can increase adherence to mobile interventions for weight loss, physical activity, safe driving, smoking cessation, among others (e.g. Patel, Volpp & Asch, 2018, NEJM; Patel et al., 2017, JAMA; Volpp et al., JAMA Internal Med). The productive team at Way to Health allows for quick development while maintaining a high level of rigor. All of our timeline goals as we have developed this projects have been met by the University of Pennsylvania team so we are confident that we will have timely delivery of the mobile application.

3. Subject Selection

3.1 Study Population

Subjects between 18 and 25 with a diagnosis of an anxiety disorder who meet the inclusion and exclusion criteria will be eligible for participation in this study.

3.2 Inclusion Criteria

Individuals who are between the ages of 18 and 25 and who have a primary diagnosis of an Anxiety Disorder, as determined by a score of 4 on the Clinical Severity Rating from the Anxiety Disorders Interview Schedule (ADIS), will be considered eligible to participate in this study.

3.3 Exclusion Criteria

Individuals who do not meet the cutoff score on the ADIS or who have a primary diagnosis other than an anxiety disorder will be excluded from participating in this study. Additionally, participants who are currently in cognitive behavioral therapy outside of the app or who have changed their dose of a psychiatric medication in the past 12 weeks will be excluded. Participants with the intent or plan to attempt suicide will be excluded from this study and provided referrals for treatment.

4. Registration Procedures

4.1 Patient Registration

Subjects will be registered within the WRG-CT as per the standard operating procedure for Subject Registration.

4.2 Study Procedures

4.3 Schedule of Evaluations

4.3.1 Screening Visit

- Anxiety Disorders Interview Schedule (ADIS) to get a Clinical Severity Rating (CSR must be 4 or higher to receive a primary diagnosis of Anxiety Disorder, and no other psychiatric conditions can have a higher CSR). This assessment will take place either in person, over the phone, or on a HIPAA-compliant virtual meeting platform. Decisions about how visits will take place will be made with participants, depending on safety and convenience.
- We will collect contact information so that we can follow up with the participant after the evaluation.
- We will also collect medication and psychiatric treatment information

4.3.2 Treatment Phase

All study visits will take place either in person, over the phone, or on a HIPAA-compliant virtual meeting platform. Decisions about how visits will take place will be made with participants, depending on safety and convenience.

4.3.2.1 Visit 1

- Participants are randomized to one of three incentive conditions (gain-framed points, loss-framed points, or social support). Participants in the social support condition will identify a person in their life who will receive text message updates about their progress—or they can choose to have the study team receive the updates.
- Demographic information
- Test of Premorbid Functioning for Wechsler Intelligence Scale for Adults (WAIS) unless completed within 18 months.
- Rumination Response Scale (RRS)
- Hamilton Rating Scale for Depression (HAM-D)
- Hamilton Rating Scale for Anxiety (HAM-A)
- Anxiety Sensitivity Index (ASI-3)
- Liebowitz Social Anxiety Scale (LSAS)
- Generalized Anxiety Disorder 7-item Scale (GAD-7)
- Patient Health Questionnaire 9 (PHQ-9)

- The Temporal Experience of Pleasure Scale (TEPS)
- Positive and Negative Affect Schedule (PANAS)

- Emotion Regulation Questionnaire (ERQ)
- Multidimensional Experiential Avoidance Questionnaire (MEAQ)
- Pittsburgh Sleep Quality Index (PSQI)
- World Health Organization Quality of Life short-form scale (WHOQOL-BREF)
- Life Events Scale for Students (LESS)
- Sensitivity to Punishment and Sensitivity to Reward Questionnaire (SPSRQ)
- Multidimensional Assessment of Interoceptive Awareness (MAIA)
- Digital Working Alliance Inventory (D-WAI)
- Optional MRI Tasks: Points Incentive Delay Task, Social Incentive Delay Task
- Optional EEG
- Medication tracking

4.3.2.2 Visit 2 (+7 days from Visit 1)—over the phone or in person

- Generalized Anxiety Disorder 7-item Scale (GAD-7)
- Patient Health Questionnaire 9 (PHQ-9)
- Mobile Application Rating Scale: user version (uMARS)
- Check in about how the app is working.
- Medication tracking

4.3.2.3 Visit 3 (+7 days from Visit 2) —over the phone or in person

- Generalized Anxiety Disorder 7-item Scale (GAD-7)
- Patient Health Questionnaire 9 (PHQ-9)
- Check in about how the app is working.
- Medication tracking

4.3.2.4 Visit 4 (+7 days from Visit 3)

- Rumination Response Scale (RRS)
- Hamilton Rating Scale for Depression (HAM-D)
- Hamilton Rating Scale for Anxiety (HAM-A)
- Generalized Anxiety Disorder 7-item Scale (GAD-7)
- Patient Health Questionnaire 9 (PHQ-9)
- The Temporal Experience of Pleasure Scale (TEPS)
- Positive and Negative Affect Schedule (PANAS)
- Intolerance of Uncertainty Scale (IUS)
- Emotion Regulation Questionnaire (ERQ)
- Multidimensional Experiential Avoidance Questionnaire (MEAQ)
- Pittsburgh Sleep Quality Index (PSQI)
- World Health Organization Quality of Life short-form scale (WHOQOL-

BREF)
- Life Events Scale for Students (LESS)
- Anxiety Sensitivity Index (ASI-3)
- Liebowitz Social Anxiety Scale (LSAS)
- Sensitivity to Punishment and Sensitivity to Reward Questionnaire (SPSRQ)
- Mobile Application Rating Scale: user version (uMARS)
- Digital Working Alliance Inventory (D-WAI)
- Check in about how the app is working.
- Medication tracking

4.3.2.5 Visit 5 (+7 days from Visit 4) —over the phone or in person

- Generalized Anxiety Disorder 7-item Scale (GAD-7)
- Patient Health Questionnaire 9 (PHQ-9)
- Check in about how the app is working.
- Medication tracking

4.3.2.6 Visit 6 (+7 days from Visit 5) —over the phone or in person

- Generalized Anxiety Disorder 7-item Scale (GAD-7)
- Patient Health Questionnaire 9 (PHQ-9)
- Check in about how the app is working.
- Medication tracking

4.3.2.7 Visit 7 (+7 days from Visit 6)

- *ADIS* sections for the anxiety disorder(s) the participant met criteria for at screening
- Rumination Response Scale (RRS)
- Hamilton Rating Scale for Depression (HAM-D)
- Hamilton Rating Scale for Anxiety (HAM-A)

- Generalized Anxiety Disorder 7-item Scale (GAD-7)
- Patient Health Questionnaire 9 (PHQ-9)
- The Temporal Experience of Pleasure Scale (TEPS)
- Positive and Negative Affect Schedule (PANAS)
- Emotion Regulation Questionnaire (ERQ)
- Multidimensional Experiential Avoidance Questionnaire (MEAQ)
- Pittsburgh Sleep Quality Index (PSQI)
- World Health Organization Quality of Life short-form scale (WHOQOL-BREF)
- Life Events Scale for Students (LESS)
- Anxiety Sensitivity Index (ASI-3)
- Liebowitz Social Anxiety Scale (LSAS)

- Sensitivity to Punishment and Sensitivity to Reward Questionnaire (SPSRQ)
- Mobile Application Rating Scale: user version (uMARS)
- Digital Working Alliance Inventory (D-WAI)
- Optional MRI Tasks: MRI Tasks: Points Incentive Delay Task, Social Incentive Delay Task
- Check in about how the app is working.
- Medication tracking
- Optional EEG

4.3.2.8 Visit 8 (+42 days from Visit 7)

- Rumination Response Scale (RRS)
- Hamilton Rating Scale for Depression (HAM-D)
- Hamilton Rating Scale for Anxiety (HAM-A)
- Generalized Anxiety Disorder 7-item Scale (GAD-7)
- Patient Health Questionnaire 9 (PHQ-9)
- The Temporal Experience of Pleasure Scale (TEPS)
- Positive and Negative Affect Schedule (PANAS)

- Emotion Regulation Questionnaire (ERQ)
- Multidimensional Experiential Avoidance Questionnaire (MEAQ)
- Pittsburgh Sleep Quality Index (PSQI)
- World Health Organization Quality of Life short-form scale (WHOQOL-BREF)
- Life Events Scale for Students (LESS)
- Anxiety Sensitivity Index (ASI-3)
- Liebowitz Social Anxiety Scale (LSAS)
- Sensitivity to Punishment and Sensitivity to Reward Questionnaire (SPSRQ)
- Mobile Application Rating Scale: user version (uMARS)
- Digital Working Alliance Inventory (D-WAI)
- Check in about how the app is working.

4.4 Treatment Administration

Treatment will be administered on an *outpatient* basis.

Participants will be asked to engage with the mobile application at least 2 days a week for 20-25 minutes per session. These sessions can be done at the participants' convenience for various lengths of time. These tasks can be boring or frustrating for some people, but participants are allowed to take breaks, or to make sessions shorter. Participants may also decide to stop participating at any time.

4.5 General Concomitant Medication and Supportive Care Guidelines

Participants will likely be seeking therapeutic services, medication-based as well as psychotherapy, outside of the research context for their anxious and depressed symptoms. This will be recorded by our protocol but will not interfere with their use of the mobile application. We do not foresee any possible risks associated with using the mobile application in addition to external non-CBT therapy.

4.6 Duration of Therapy and Criteria for Removal from Study

In the absence of treatment delays due to adverse event(s), treatment may continue for 12 weeks or until one of the following criteria applies:

The study will take place for a total of 6 weeks but participants will continue to have access to the mobile device if they would like to after the study is complete. Participants are welcome to withdraw from the study at their own discretion. There are no general of specific changes in the patient's condition that would render the patient unacceptable for further treatment in the judgement of the investigator.

4.7 Duration of Follow Up

Patients will be followed for 6 weeks while they are participating in the study. There will also be a follow up appointment 6 weeks after they finish the study.

5. Measurement of Effect

We will be measuring the effects of the mobile CBT program using clinical measures that test symptoms of anxiety and depression, namely, the Hamilton Rating Scale for Depression and the Hamilton Rating Scale for Anxiety. Optional fMRI scans will also be collected. The MRI scans will be done without contrast.

6. Statistical Considerations

As this study is a pilot to determine feasibility of the CBT mobile application including non-monetary incentives, we do not anticipate high volume data. We intend to use the pilot data to submit an NIH grant where we will recruit a statistician to be a part of our research team.

6.1 Study Design/Endpoints

No subjects will be forced to end participation in the study based on a lack of engagement with the mobile CBT program. We will be looking at whether participants completed the protocol as intended (i.e. usage statistics across the 6 weeks), We will gather the qualitative data from participants when they come for the weekly ~~in-person~~ check-in

visits. We will analyze how often participants opened the mobile app, completed the exercises and whether they completed the 6 week protocol, and if not, at what time point they stopped participating. We have no set criteria regarding what percentage must finish the protocol; however, if less than 25% of participants complete the protocol as intended, we will re- evaluate our current procedures. Such information is a critical first step *before* testing the validity of the mobile CBT platform, which will be the scope of future proposals to the NIH. We will also be comparing differences in efficacy and treatment engagement between three different incentive conditions. We will not be comparing the mobile CBT to any other type of treatment at this initial stage.

6.2 Sample Size/Accrual Rate

We plan on recruiting 300 subjects over the course of a year, with the anticipation that 120 will participate in the study to completion. This means we hope to recruit approximately 25 subjects per month.

6.3 Stratification Factors

There are no planned patient stratification factors.

6.3.1 **Analysis of Endpoints**

6.3.2 **Analysis of Primary Endpoints**

The primary purpose of the study is to compare change in anxious symptoms from baseline to Visit 7 (end of treatment with the mobile cognitive behavior mobile application) between the three incentive conditions. The primary measure of this endpoint will be the Hamilton Rating Scale for Anxiety (HAM-A), a 14-item questionnaire measure of the severity of anxiety symptoms. The items measure both psychic anxiety and somatic anxiety, where higher scores indicate a greater presence of symptoms and lower scores indicate mild to no anxiety symptoms. Secondary measures of this endpoint will be the Anxiety Sensitivity Index (ASI) and the Leibowitz Social Anxiety Scale (LSAS). The ASI is an 18-item scale containing three subscales measuring physical, cognitive, and social concerns regarding anxiety. Higher scores reflect greater self-reported concern and lower scores reflect little self-reported concern. The LSAS is a 24-item scale measuring fear and avoidance across social situations. Higher scores indicate greater fear and avoidance severity and lower scores indicate mild to no fear or avoidance.

6.3.3 **Analysis of Secondary Endpoints**

A secondary aim of the study is to compare participants' engagement with the mobile app between the three incentive conditions. The primary measure of this endpoint is the number of sessions completed over the course of treatment. Secondary

measures are the total time spent engaging with the app during the course of treatment and participants' impressions of the program as assessed by the Mobile Application Rating Scale (uMARS). The uMARS is a 26-item measure of mobile application engagement, functionality, aesthetics, quality of information, and perceived impact. Higher scores indicate greater application feasibility and lower scores indicate poorer application feasibility.

References

Albanese, B. J., Macatee, R. J., Stanley, I. H., Bauer, B. W., Capron, D. W., Bernat, E., ... & Schmidt, N. B. (2019). Differentiating suicide attempts and suicidal ideation using neural markers of emotion regulation. *Journal of affective disorders*, *257*, 536-550.

O. Remes, C. Brayne, R. van der Linde, L. Lafortune. A systematic review of reviews on the prevalence ofanxiety disorders in adult populations, *Brain and Behavior*, 2016; 6(7), e00497, doi: 10.1002/brb3.497

Schupp, H., Cuthbert, B., Bradley, M., Hillman, C., Hamm, A., & Lang, P. (2004). Brain processes in emotional perception: Motivated attention. *Cognition and emotion*, *18*(5), 593-611.

Weinberg, A., & Hajcak, G. (2010). Beyond good and evil: the time-course of neural activity elicited by specific picture content. *Emotion*, *10*(6), 767.

Weinberg, A., Perlman, G., Kotov, R., & Hajcak, G. (2016). Depression and reduced neural response to emotional images: Distinction from anxiety, and importance of symptom dimensions and age of onset. Journal of abnormal psychology, 125(1), 26.

Sabatinelli, D., Keil, A., Frank, D. W., & Lang, P. J. (2013). Emotional perception: correspondence of early and late event-related potentials with cortical and subcortical functional MRI. *Biological psychology*, *92*(3), 513-519.
