# Supplementary material for: Efficacy of a Mobile App-Based Intervention for Young Adults With Anxiety Disorders: A Randomized Clinical Trial
Source: JAMA Netw Open. 2024 Aug 20;7(8):e2428372. doi: 10.1001/jamanetworkopen.2024.28372 (PMC11337073; doi:10.1001/jamanetworkopen.2024.28372)
Supplement: Supplement 2. — eFigure. Therapeutic Themes, Symptom Targets, and Modules Included at Each Week of the Maya Program eMethods. Descriptions of Measures and Training Procedures eResults. Linear Mixed Model Results by Outcome eDiscussion. Supplementary Discussion eReferences [file jamanetwopen-e2428372-s002.pdf]

Supplementary Online Content

Bress JN, Falk A, Schier MM, et al. Efficacy of an app-based intervention for young adults with anxiety disorder: a randomized clinical trial. *JAMA Netw Open*. 2024;7(8):e2428372. doi:10.1001/jamanetworkopen.2024.28372

eFigure. Therapeutic Themes, Symptom Targets, and Modules Included at Each Week of the Maya Program

eMethods. Descriptions of Measures and Training Procedures

eResults. Linear Mixed Model Results by Outcome

eDiscussion. Supplementary Discussion

eReferences

This supplementary material has been provided by the authors to give readers additional information about their work.

© 2024 Bress JN et al. *JAMA Network Open.*

eFigure. Therapeutic Themes, Symptom Targets, and Modules Included at Each Week of the Maya Program

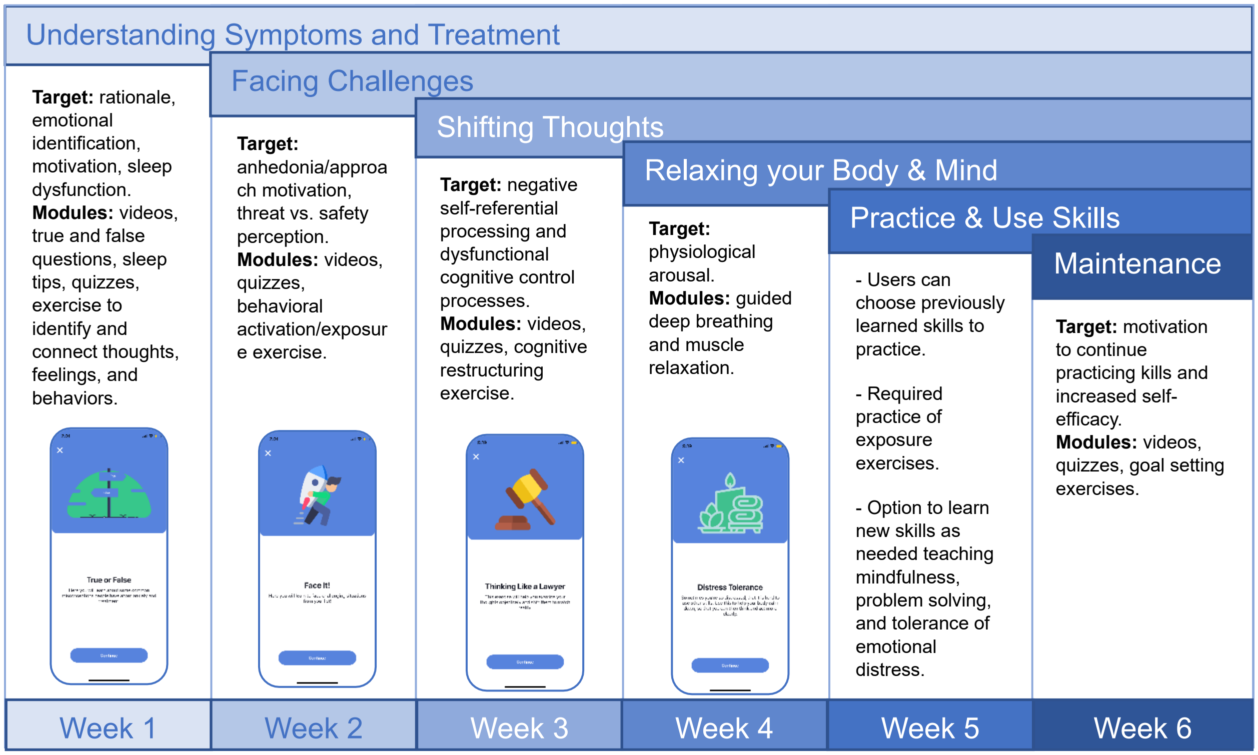

© 2024 Bress JN et al. *JAMA Network Open.*

eMethods. Descriptions of Measures and Training Procedures

Diagnostic Assessment

The ADIS-5[1] was administered to determine psychiatric diagnosis and clinical severity rating (CSR) at baseline and endpoint. The ADIS-5 is a semi-structured interview designed to assess current and past anxiety, mood, obsessive-compulsive, trauma, and related disorders in adults, and to screen for other disorders (e.g., eating and psychotic disorders) with strong psychometric properties[2]. A senior study clinician reviewed all ADIS assessments to confirm the diagnosis. If there were any questions regarding the correct diagnosis, they were addressed by consensus conference with a team of four clinical psychologists including the senior and first authors, all of whom had a minimum of six years of clinical experience.

Efficacy

Primary Outcome: Anxiety. The HAM-A was used as the primary measure of anxiety. The HAM-A is a clinician-administered 14-item measure of the severity of anxiety symptoms that has demonstrated adequate reliability and concurrent validity[3,4]. The items measure both psychic and somatic anxiety, with a total score range of 0-56. Scores of ≤ 7 indicate no/minimal anxiety, 8-14 indicate mild anxiety, 15-23 indicate moderate anxiety, and ≥ 24 indicate severe anxiety[5].

The ASI[6] was used as a secondary measure of anxiety. The ASI consists of 18 self-report items across three domains (physiological, cognitive, and social concerns) assessing fear of the consequences of experiencing anxiety (anxiety sensitivity, or "fear of fear"). Scores range from 0-72, with higher scores indicating greater anxiety sensitivity. The ASI demonstrates good psychometric properties[6].

The LSAS[7] was also used as a secondary measure of anxiety. The LSAS is a 24-item self-report rating scale measuring social anxiety, with a total score range of 0-144. The LSAS demonstrates strong validity[8].

Exploratory Outcome: Depression. Depressive symptoms were measured with the 24-item HAM-D[9]. The HAM-D has good reliability and validity and is one of the most widely used clinician-administered depression scales[9,10]. The 24-item HAM-D has a total score range of 0-76, with ≤ 7 indicating absence of depression, 8-14 indicating borderline to mild depression, 15-25 indicating moderate to marked depression, and ≥ 26 indicating severe to extreme depression.

Engagement and App Satisfaction

Engagement. Engagement with the app was measured in terms of the total number of app sessions completed out of the 12 assigned. Retention was assessed at week 6 (end of intervention) and at the week 12 follow-up.

Participant Satisfaction. Participant satisfaction was measured with the uMARS[11]. The uMARS is a 20-item measure of satisfaction with an app; 16 of the items are used to calculate an "App Quality" score along the following dimensions: engagement (how interesting, fun, and interactive the app is), functionality (ease of use, general design), aesthetics (layout, graphics, visual appeal), and information conveyed (quality, quantity, credibility of content). Each item is rated on a 5-point Likert-type scale. The uMARS total App Quality score is the mean score across these 16 items, where a mean score of 4 or greater denotes good satisfaction and a score of 3 or lower denotes poor satisfaction. The uMARS has strong internal consistency and acceptable test-retest reliability[11].

Training Procedures

Raters were blinded to group assignment. Study staff administering interview-based measures (ADIS, HAM-A, HAM-D) were trained by licensed clinical psychologists and were required to demonstrate agreement on scores (within 1 point on each item, and agreement on whether ADIS diagnoses were clinically significant) on at least three assessments prior to administering each measure independently.

Exploratory Analyses

Efficacy of Maya on Depression

To evaluate the effect of Maya on the exploratory depression outcome (HAM-D), we used linear mixed effects models with subject intercept as a random effect. All subjects with baseline data were included following an intent-to-treat approach. We tested the main effect of time (Baseline [Week 0], Midpoint [Week 3], Endpoint [Week 6], and Follow-up [Week 12]) as a fixed effect, incentive condition (Social Support, Gain Framed, Loss Framed) as a fixed effect, and a time x incentive condition interaction.

Efficacy of Maya by Baseline Symptom Severity

We conducted post-hoc analyses exploring whether the efficacy of Maya differed by baseline symptom severity. We collapsed data across incentive conditions and computed separate linear mixed effects models for each outcome measure with subjects as a random effect, time as a fixed effect, baseline severity score (on the HAM-A, ASI, LSAS, and HAM-D respectively) and the time x baseline severity score interaction.

© 2024 Bress JN et al. *JAMA Network Open.*

eResults. Linear Mixed Model Results by Outcome

Hamilton Anxiety Rating Scale

Mixed Model

Model Info

Info	
Estimate	Linear mixed model fit by REML
Call	Ham-A Score ~ 1 + Time + Group + Time:Group+(1 | subj)
AIC	1396.277
BIC	1419.111
LogLikel.	−671.489
R-squared Marginal	0.205
R-squared Conditional	0.596
Converged	yes
Optimizer	bobyqa

[3]

Model Results

Fixed Effect Omnibus tests

	F	Num df	Den df	p
Time	21.58	3	163.4	<.001
Group	1.71	2	56.5	0.190
Time ✻ Group	1.15	6	163.2	0.336

Note. Satterthwaite method for degrees of freedom

Random Components

Groups	Name	SD	Variance	ICC
subj	(Intercept)	3.92	15.4	0.493
Residual		3.98	15.9	

Note. Number of Obs: 230 , groups: subj 59

Anxiety Sensitivity Index

Mixed Model

Model Info

Info	
Estimate	Linear mixed model fit by REML
Call	ASI_Total ~ 1 + Time + Group + Time:Group+(1 | subj)
AIC	1674.333
BIC	1682.722
LogLikel.	−803.294
R-squared Marginal	0.114
R-squared Conditional	0.776
Converged	yes
Optimizer	bobyqa

[3]

Model Results

Fixed Effect Omnibus tests

	F	Num df	Den df	p
Time	23.195	3	162.1	<.001
Group	0.732	2	55.7	0.486
Time ✻ Group	0.777	6	162.0	0.589

Note. Satterthwaite method for degrees of freedom

© 2024 Bress JN et al. *JAMA Network Open.*

Random Components

Groups	Name	SD	Variance	ICC
subj	(Intercept)	11.09	123.0	0.748
Residual		6.44	41.5	

Note. Number of Obs: 230 , groups: subj 59

Leibowitz Social Anxiety Scale

Mixed Model

Model Info

Info	
Estimate	Linear mixed model fit by REML
Call	Total LSAS Score ~ 1 + Time + Group + Time:Group+(1 | subj)
AIC	1947.151
BIC	1940.791
LogLikel.	−932.360
R-squared Marginal	0.120
R-squared Conditional	0.784
Converged	yes
Optimizer	bobyqa

[3]

>

Model Results

Fixed Effect Omnibus tests

	F	Num df	Den df	p
Time	19.066	3	161.4	<.001
Group	1.790	2	56.0	0.176
Time ✻ Group	0.389	6	161.3	0.885

Note. Satterthwaite method for degrees of freedom

Random Components

Groups	Name	SD	Variance	ICC
subj	(Intercept)	20.7	430	0.755
Residual		11.8	139	

Note. Number of Obs: 229 , groups: subj 59

© 2024 Bress JN et al. *JAMA Network Open.*

Efficacy of Maya on Depression

There was a significant main effect of time on depression severity (HAM-D). Post-hoc tests revealed that depression severity was significantly lower at midpoint (mean difference = -2.91, 95% CI [-4.58, -1.23], Cohen's d = 0.51), endpoint (mean difference = -5.14, 95% CI [-6.84, -3.44], Cohen's d = 0.81), and follow-up (mean difference = -6.56, 95% CI [-8.30, -4.82], Cohen's d = 1.03) compared to baseline. There was no main effect of incentive condition, and no statistically significant time x incentive condition interaction, on HAM-D score.

In a planned exploratory analysis, to further characterize the potential effect of comorbid depression, we computed linear mixed effects models on HAM-A, ASI, and LSAS and evaluated a time x major depressive disorder diagnosis (yes or no, based on the ADIS) interaction. Linear mixed effects models did not reveal significant time x depression diagnosis interactions for the HAM-A, ASI, or LSAS, suggesting that improvement over time on these measures was not moderated by MDD diagnosis (all F's < 2.22, all p's > .05).

Mixed Model

Model Info

Info	
Estimate	Linear mixed model fit by REML
Call	Total Ham-D Score ~ 1 + Time + Group + Time:Group+(1 | subj)
AIC	1428.241
BIC	1449.407
LogLikel.	−686.637
R-squared Marginal	0.158
R-squared Conditional	0.574
Converged	yes
Optimizer	bobyqa

[3]

Model Results

Fixed Effect Omnibus tests

	F	Num df	Den df	p
Time	21.071	3	163.8	<.001
Group	0.619	2	56.9	0.542
Time ✻ Group	0.429	6	163.6	0.859

Note. Satterthwaite method for degrees of freedom

Fixed Effects Parameter Estimates

Names	Effect	Estimate	SE	95% Confidence Interval Lower	95% Confidence Interval Upper	df	t	p
(Intercept)	(Intercept)	11.929	0.672	10.61	13.25	57.1	17.741	<.001
Time1	Midpoint - Baseline	−2.906	0.853	−4.58	−1.23	162.6	−3.407	<.001
Time2	Endpoint - Baseline	−5.141	0.867	−6.84	−3.44	163.7	−5.928	<.001
Time3	Followup - Baseline	−6.560	0.888	−8.30	−4.82	164.6	−7.383	<.001
Group1	Loss-Framed - Gain-Social	−1.417	1.822	−4.99	2.15	57.2	−0.778	0.440
Group2	Gain-Framed - Gain-Social	0.454	1.411	−2.31	3.22	56.0	0.322	0.749
Time1 ✻ Group1	Midpoint - Baseline ✻ Loss-Framed - Gain-Social	0.838	2.310	−3.69	5.36	162.6	0.363	0.717
Time2 ✻ Group1	Endpoint - Baseline ✻ Loss-Framed - Gain-Social	−1.833	2.357	−6.45	2.79	163.9	−0.778	0.438
Time3 ✻ Group1	Followup - Baseline ✻ Loss-Framed - Gain-Social	1.650	2.410	−3.07	6.37	164.8	0.685	0.495
Time1 ✻ Group2	Midpoint - Baseline ✻ Gain-Framed - Gain-Social	−0.222	1.799	−3.75	3.30	162.6	−0.124	0.902
Time2 ✻ Group2	Endpoint - Baseline ✻ Gain-Framed - Gain-Social	−0.756	1.799	−4.28	2.77	162.6	−0.420	0.675
Time3 ✻ Group2	Followup - Baseline ✻ Gain-Framed - Gain-Social	0.838	1.822	−2.73	4.41	163.1	0.460	0.646

Random Components

Groups	Name	SD	Variance	ICC
subj	(Intercept)	4.22	17.8	0.494
Residual		4.27	18.2	

Note. Number of Obs: 230 , groups: subj 59

© 2024 Bress JN et al. *JAMA Network Open.*

Efficacy of Maya by Baseline Symptom Severity

Separate linear mixed effects models revealed a significant time x baseline score interaction for the HAM-A (F(3, 167.2)=18.3, p<.001), ASI (F(3, 168.3)=18.6), p<.001, LSAS (F(3, 165.5)=12.3, p<.001), and HAM-D (F(3, 166.8)=11.4, p<.001). Those with higher baseline scores on the HAM-A, ASI, LSAS, and HAM-D demonstrated a steeper slope of change (improvement) in scores than those with low baseline scores on each measure.

Figure S2. Results of the linear mixed effects model demonstrating change in outcome measure by time and baseline score (severity) on each measure. For visualization purposes, the plots display the group means estimated by the model at three different levels of the covariate (baseline score): mean, mean + 1 SD, and mean – 1 SD.

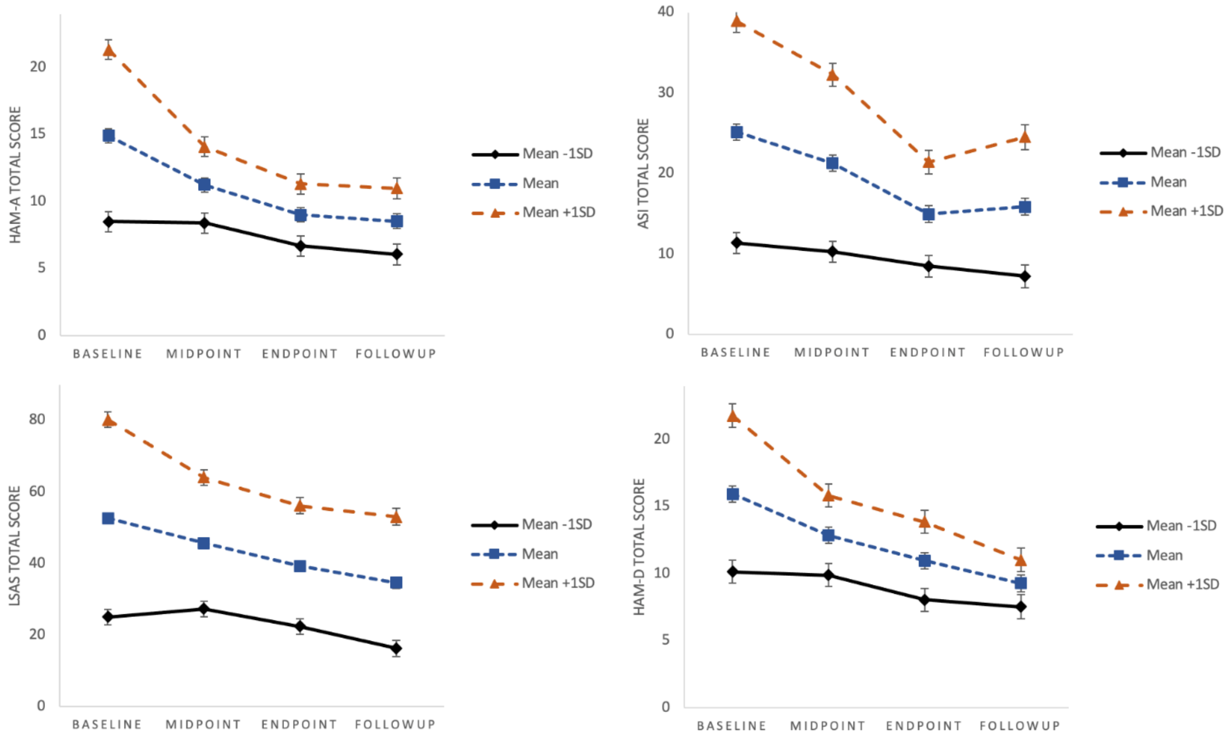

© 2024 Bress JN et al. *JAMA Network Open.*

eDiscussion. Supplementary Discussion

Depression

Depression decreased over the course of the intervention, and improvement of depression was maintained at follow-up. Although the sample was selected for anxiety rather than depression, mean depression scores were in the moderate range of severity[9], and 20% of the sample had a comorbid diagnosis of MDD or another depressive disorder. Maya includes modules highly relevant to depression, which target symptoms including negative self-referential thinking, behavioral withdrawal, and inadequate sleep hygiene. Therefore, the current results provide preliminary evidence that emerging adults with anxiety and comorbid symptoms of depression may benefit from Maya to a similar degree as those with anxiety alone.

Baseline Symptom Severity

The finding that efficacy was greater for participants with higher baseline anxiety severity is consistent with prior studies[12–15]. Although regression to the mean cannot be ruled out, this finding may reflect greater capacity for change with use of Maya among individuals with more severe anxiety. It also suggests the potential usefulness of self-guided mental health apps in more acute treatment settings, even for those with more significant psychopathology. Indeed, while a majority of patients in an acute psychiatric care setting express interest in mental health apps, few have downloaded apps focusing on therapy skills[16], highlighting a role for such interventions. However, as all participants in the current study were outpatients without significant suicidal ideation, and mean anxiety was in the mild to moderate range, understanding the utility of Maya in more severely symptomatic populations will require further study.

© 2024 Bress JN et al. *JAMA Network Open.*

eReferences

1. Brown TA, Barlow DH. *Anxiety and Related Disorders Interview Schedule for DSM-5 (ADIS-5): Client Interview Schedule*. Adult version. Oxford University Press; 2014.
2. Brown TA, Di Nardo PA, Lehman CL, Campbell LA. Reliability of DSM-IV anxiety and mood disorders: Implications for the classification of emotional disorders. *Journal of Abnormal Psychology*. 2001;110(1):49-58. doi:10.1037/0021-843X.110.1.49
3. Hamilton M. The Assessment of Anxiety States by Rating. *British Journal of Medical Psychology*. 1959;32(1):50-55. doi:10.1111/j.2044-8341.1959.tb00467.x
4. Maier W, Buller R, Philipp M, Heuser I. The Hamilton Anxiety Scale: reliability, validity and sensitivity to change in anxiety and depressive disorders. *Journal of Affective Disorders*. 1988;14(1):61-68. doi:10.1016/0165-0327(88)90072-9
5. Matza LS, Morlock R, Sexton C, Malley K, Feltner D. Identifying HAM-A cutoffs for mild, moderate, and severe generalized anxiety disorder. *International Journal of Methods in Psychiatric Research*. 2010;19(4):223-232. doi:10.1002/mpr.323
6. Taylor S, Zvolensky MJ, Cox BJ, et al. Robust dimensions of anxiety sensitivity: Development and initial validation of the Anxiety Sensitivity Index-3. *Psychological Assessment*. 2007;19(2):176-188. doi:10.1037/1040-3590.19.2.176
7. Liebowitz MR. Social Phobia. In: Klein DF, ed. *Modern Trends in Pharmacopsychiatry*. Vol 22. S. Karger AG; 1987:141-173. doi:10.1159/000414022
8. Heimberg RG, Horner KJ, Juster HR, et al. Psychometric properties of the Liebowitz Social Anxiety Scale. *Psychol Med*. 1999;29(1):199-212. doi:10.1017/S0033291798007879
9. Hamilton M. A rating scale for depression. *J Neurol Neurosurg Psychiatry*. 1960;23(1):56-62. doi:10.1136/jnnp.23.1.56
10. Bagby RM, Ryder AG, Schuller DR, Marshall MB. The Hamilton Depression Rating Scale: Has the Gold Standard Become a Lead Weight? *AJP*. 2004;161(12):2163-2177. doi:10.1176/appi.ajp.161.12.2163
11. Stoyanov SR, Hides L, Kavanagh DJ, Wilson H. Development and Validation of the User Version of the Mobile Application Rating Scale (uMARS). *JMIR mHealth and uHealth*. 2016;4(2):e5849. doi:10.2196/mhealth.5849
12. Areán PA, Hallgren KA, Jordan JT, et al. The Use and Effectiveness of Mobile Apps for Depression: Results From a Fully Remote Clinical Trial. *Journal of Medical Internet Research*. 2016;18(12):e6482. doi:10.2196/jmir.6482
13. Niles AN, Axelsson E, Andersson E, et al. Internet-based cognitive behavior therapy for depression, social anxiety disorder, and panic disorder: Effectiveness and predictors of response in a teaching clinic. *Behaviour Research and Therapy*. 2021;136:103767. doi:10.1016/j.brat.2020.103767
14. van Straten A, Cuijpers P, Smits N. Effectiveness of a Web-Based Self-Help Intervention for Symptoms of Depression, Anxiety, and Stress: Randomized Controlled Trial. *Journal of Medical Internet Research*. 2008;10(1):e954. doi:10.2196/jmir.954
15. Wright JH, Owen JJ, Richards D, et al. Computer-Assisted Cognitive-Behavior Therapy for Depression: A Systematic Review and Meta-Analysis. *J Clin Psychiatry*. 2019;80(2):3573. doi:10.4088/JCP.18r12188
16. Beard C, Silverman AL, Forgeard M, Wilmer MT, Torous J, Björgvinsson T. Smartphone, Social Media, and Mental Health App Use in an Acute Transdiagnostic Psychiatric Sample. *JMIR Mhealth Uhealth*. 2019;7(6):e13364. doi:10.2196/13364

© 2024 Bress JN et al. *JAMA Network Open.*
