# Supplementary material for: Efficacy of a Mobile App-Based Intervention for Young Adults With Anxiety Disorders: A Randomized Clinical Trial
Source: JAMA Netw Open. 2024 Aug 20;7(8):e2428372. doi: 10.1001/jamanetworkopen.2024.28372 (PMC11337073; doi:10.1001/jamanetworkopen.2024.28372)
Supplement: Supplement 3. — Data Sharing Statement [file jamanetwopen-e2428372-s003.pdf]

# Data Sharing Statement

Bress. Efficacy of a Mobile App-Based Intervention for Young Adults With Anxiety Disorders. *JAMA Netw Open*. Published August 20, 2024. doi:10.1001/jamanetworkopen.2024.28372

**Data**
**Data available:** Yes
**Data types:** Deidentified participant data, Data dictionary
**How to access data:** requests can be sent to fgd2002@med.cornelll.edu
**When available:** With publication

**Supporting Documents**
**Document types:** None

**Additional Information**
**Who can access the data:** Data will be made available to researchers whose proposed use of the data has been approved.
**Types of analyses:** Data will be made available for analyses for a specified purpose.
**Mechanisms of data availability:** Data will be made available both after approval of a proposal and with a signed data access agreement.
